# Cooperation among unrelated ant queens provides persistent growth and survival benefits during colony ontogeny

**DOI:** 10.1038/s41598-021-87797-5

**Published:** 2021-04-15

**Authors:** Madeleine M. Ostwald, Xiaohui Guo, Tyler Wong, Armon Malaekeh, Jon F. Harrison, Jennifer H. Fewell

**Affiliations:** School of Life Sciences, Arizona State University, Tempe, AZ USA

**Keywords:** Behavioural ecology, Social evolution

## Abstract

The fitness consequences of cooperation can vary across an organism’s lifespan. For non-kin groups, especially, social advantages must balance intrinsic costs of cooperating with non-relatives. In this study, we asked how challenging life history stages can promote stable, long-term alliances among unrelated ant queens. We reared single- and multi-queen colonies of the primary polygynous harvester ant, *Pogonomyrmex californicus*, from founding through the first ten months of colony growth, when groups face high mortality risks. We found that colonies founded by multiple, unrelated queens experienced significant survival and growth advantages that outlasted the colony founding period. Multi-queen colonies experienced lower mortality than single-queen colonies, and queens in groups experienced lower mortality than solitary queens. Further, multi-queen colonies produced workers at a faster rate than did single-queen colonies, even while experiencing lower per-queen worker production costs. Additionally, we characterized ontogenetic changes in the organization of labor, and observed increasing and decreasing task performance diversity by workers and queens, respectively, as colonies grew. This dynamic task allocation likely reflects a response to the changing role of queens as they are increasingly able to delegate risky and costly tasks to an expanding workforce. Faster worker production in multi-queen colonies may beneficially accelerate this behavioral transition from a vulnerable parent–offspring group to a stable, growing colony. These combined benefits of cooperation may facilitate the retention of multiple unrelated queens in mature colonies despite direct fitness costs, providing insight into the evolutionary drivers of stable associations between unrelated individuals.

## Introduction

Cooperation is a major adaptive strategy for coping with challenging life history stages^1,2^. Transient grouping can provide survival advantages during risky or vulnerable periods^1–4^. Less frequently, long-term alliances may arise when benefits of cooperation outweigh intrinsic direct fitness costs of shared reproduction^1,5–7^. For eusocial groups, as for individual organisms, early growth and development represents a particularly difficult life history stage, because young colonies are highly vulnerable to predation, mortality risks, and the effects of competition^8–10^. Simultaneously, incipient colonies must coordinate major organizational changes that support rapid growth^9,11,12^. Understanding conditions during this challenging ontogenetic period may provide insights into the social advantages underlying cooperation among colony foundresses.

For eusocial groups, cooperation among reproductive foundresses (pleometrosis) can facilitate survival and growth when risk of colony mortality is high^13–15^. Because queens in these associations are often unrelated, strong direct survival and/or productivity benefits should theoretically compensate for intrinsic fitness costs of cooperating with non-relatives^16,17^ (but see^18^). During the founding stage, shared labor may reduce risks and costs associated with essential tasks such as foraging and nest construction^19–21^. Indeed, pleometrosis has been associated with higher survival of queens^13,14,21,22^ as well as faster initial worker production^23–25^, which facilitates quick progression through the risky founding stage.

However, these benefits of cooperation may decrease or disappear as the colony matures. For the majority of pleometrotic ant species, foundress cooperation ceases immediately following the emergence of workers, after which only a single queen will survive fighting among foundresses and/or culling by workers^26,27^. In some populations, however, unrelated queen associations will persist throughout the lifespan of the colony, a condition known as primary polygyny^26–28^. This strategy has been associated with success in harsh and/or competitive environments^7,29–31^, suggesting the adaptive value of queen cooperation where risk of colony mortality is high. However, we lack experimental evidence linking group and individual survival benefits to long-term maintenance of queen cooperation through the critical phase of transition between pleometrosis and primary polygyny.

Behavioral changes, both by queens and workers, are likely to accompany the transition from founding to early colony ontogeny. Soon after founding, female reproductives must navigate the behavioral transition from their role as a mother rearing offspring (a foundress) to that of the reproductive head of a colony (a queen). During colony founding, foundresses initiate work essential to group survival and growth prior to the emergence of a functional colony workforce, especially those that must forage to support the development of their first brood^27,32,33^. In mature colonies, however, most queens specialize on egg production and contribute little, if at all, to non-reproductive tasks^34^. The expectation that queens cease work immediately following first-worker emergence belies the complexity of this developmental transition and the need for dynamic task allocation programs that accommodate rapid shifts in workforce capabilities. Instead, like most ontogenetic processes, it is likely that this transition features gradual organizational shifts that facilitate the scaling of labor through flexibility of behavioral roles.

Workers, likewise, must dynamically tailor their behavior to the ontogenetic stage of the colony. Shifts in the organization of work likely accommodate changes in developmental status by prioritizing relevant tasks. For example, workers in small harvester ant colonies (*Pogonomyrmex californicus*) tend to perform relatively more brood care than they do in larger colonies, likely to maximize investment in growth^35^. Simultaneously, colonies increasingly allocate labor toward waste management and food processing as they grow, as the need for colony maintenance increases with size^35^. As colonies grow in size, their workforce can become increasingly specialized, both morphologically and behaviorally^33,36–41^. These studies have elucidated the organizational mechanisms supporting the pre-reproductive growth stage of the colony life cycle^35,38,42,43^, but the earlier ontogenetic processes at the birth of the colony remain to be examined.

We asked how queen cooperation impacts growth and survival during the vulnerable phase of early colony ontogeny in a facultatively primary polygynous harvester ant, *P. californicus*. This species exhibits population-level variation in nest founding strategies, wherein nests may be founded solitarily or by multiple unrelated queens^22,44^. Queens in primarily polygynous populations of this species have reduced reproductive output relative to monogynous populations^7^, suggesting that cooperation may be a response to survival or growth constraints, likely during the challenging phase of colony ontogeny. We tracked colony survival and growth during founding and through the first ten months of colony growth. Further, we examined behavioral signatures of this developmental stage by measuring changes in queen and worker task allocation as colonies grew. While pleometrosis confers important survival benefits during colony founding^22^, at colony maturity queens may experience direct reproductive costs of cooperation with non-relatives^45^. By linking these two life history stages, exploring conditions at early colony ontogeny may provide essential insights into the benefits underlying sustained cooperation among non-kin.

## Methods

### Queen collection and colony maintenance

To characterize the founding and early growth of single- and multi-queen harvester ant colonies, we collected newly-mated *P. californicus* foundresses following mating flights from a known majority pleometrotic (cooperative founding) population in Pine Valley, San Diego County, California (32°49′20″ N, 116°31′43″ W, 1136 m elevation) in June 2018. Foundresses were randomly assigned to nests either singly, in groups of two, or in groups of four foundresses (*N* = 30 nests for each foundress number condition). Foundress associations at this site consist of an average of 4.1 individuals, with rare single-queen colonies^7^. Thus, our two- and four-foundress conditions approximated natural conditions, while our single-foundress condition served to illustrate by comparison the effects of queen cooperation on early colony survival and growth. Nests consisted of two plastic chambers (9 cm diameter, 3.5 cm height) joined by vinyl tubing, with one closed chamber containing a water reservoir and simulating the nest, and the second, open chamber simulating a foraging arena. Colonies were maintained at 30 ± 1 °C and exposed to ambient day-night light conditions for the lab location (Tempe, AZ, USA: 33°25′28″ N, 111°55′41″ W). The foraging arena was supplied with ad libitum Kentucky bluegrass and sesame seeds (1:1 by vol) and once weekly with fruit flies (*Drosophila melanogaster*) or an agar-based ant diet^46^.

### Colony growth and survival analysis

To assess early colony growth and survival, colonies were censused once weekly from initial colony founding in June 2018 through November 2018, then biweekly through April 2019. We censused more frequently during the first five months following colony founding to obtain higher-resolution estimates of growth rates during the period of rapid colony growth. During each census, we made counts of surviving queens, workers, and colonies. Surviving colonies were defined as those with at least one living queen. Only colonies with no queen death at the time of sampling were included in worker counts and in the calculation of per-queen worker production.

### Behavioral analysis

To characterize behavioral changes during early colony ontogeny, we conducted weekly sets of behavioral scan samples from colony founding until colonies contained 10 adult workers. We selected this sampling range because task allocation in *P. californicus* is well-characterized for foundress groups^47^ and for colonies containing 10 or more workers^35,38^, but the critical transition phase in between remains poorly understood. All colonies reached a size of 10 workers between week 7 and week 29, and total sampling duration depended on growth rate to 10 workers. On average, single-queen colonies reached 10 workers by week 27, two-queen colonies by week 15, and four-queen by week 11. Only colonies that reached 10 workers without any queen death were included in behavioral analyses (*N* = 42 colonies; 3 1-queen colonies, 22 2-queen colonies, 17 4-queen colonies). Each week we conducted four morning scan samples, approximately 20–30 min apart and on the same morning, of all workers and queens in each colony, such that each individual ant was observed four times every sampling day. Ants were categorized as queens or workers but not otherwise individually distinguished or marked. We recorded the behavioral state of all individuals at the time of scan sampling, and assigned all behaviors to eight major categories:

*Brood care* laying an egg (queens only); antennating/contacting brood; standing within one body-length of brood; carrying brood.

*Social interaction* antennating another; allogrooming; receiving aggression; performing aggression.

*Idle* standing/unmoving.

*Colony maintenance* removal of waste material from the nest.

*Self-maintenance* self-grooming; drinking from water reservoir.

*Walking* walking in nest chamber but not otherwise engaged in a defined task.

*Food processing*: chewing/processing seed, fruit fly, or bhatkar.

*Foraging* retrieving seed, fruit fly, or bhatkar from foraging arena; walking in foraging arena.

### Statistical analysis of colony survival and growth

To assess the effect of queen number on colony growth, we used a generalized linear mixed model (GLMM) with negative binomial distribution, with worker number as a response variable, time since first worker production, queen number, and the interaction between them as fixed effects, and colony as a random effect. We similarly used a GLMM with gamma distribution to assess individual queen worker production across queen numbers, using per-queen worker production as a response variable, time since first worker production and queen number as fixed effects, and colony as a random effect. We calculated per-queen worker production by dividing whole-colony worker number by the number of queens (1, 2, or 4) and transformed the data by adding 0.01 to each value to enable analysis of 0 values. For both analyses, we obtained estimates using type III SS with the ‘Anova’ function in the package “car”^48^, and used post-hoc Tukey tests to assess differences in growth across queen numbers. Models were fitted using the “lme4”^49^ package.

To assess colony survival across queen number treatments, we conducted log-rank survival analyses followed by post-hoc Tukey tests with Bonferroni corrections for multiple comparisons. To assess queen survival across queen number treatments, we performed mixed effect cox regression using colony as a random effect, and post-hoc Tukey tests. We report *P-*values with Bonferroni corrections for multiple comparisons. All statistical analyses were performed in R version 4.0.2 (R Development Core Team 2020). Colony survival analysis was conducted using the “survival”^50^ package and queen survival analysis was conducted using the packages “coxme”^51^ and “multcomp”^52^.

### Statistical analysis of behavioral changes during ontogeny

To assess the effect of increasing worker number on the performance of various tasks by workers or queens, we used generalized linear mixed models (GLMM) with a binomial error distribution and logit link function. For each behavioral category, we calculated the proportion of observations of that behavior per scan sample across all queens or all workers in a colony on a given sampling day (e.g., when observing two queens in one colony, two instances of brood care across eight observations = 0.25). We then constructed GLMMs for each behavioral category with this proportion as the binomial response variable, worker number as a fixed effect, and colony as a random effect. We report *P-*values with Bonferroni corrections for multiple comparisons. There was no effect of queen number nor the interaction between queen number and worker number when these were included as fixed effects, so we omitted them from the models (Supplementary Table 1). We confirmed homoscedasticity of data for each model by plotting fitted values versus residuals.

**Table 1:** Changes in behavior of queen and workers from colony founding until colonies contained 10 workers, analyzed by GLMM.

Queens			Workers		
Behavior	*P*-value	Slope (change in incidence/worker number; mean ± std. error)	Behavior	*P*-value	Slope (change in incidence/worker number; mean ± std. error)
Foraging	< 0.001	− 0.544 ± 0.077	Foraging	< 0.001	0.134 ± 0.018
Idle	< 0.001	− 0.120 ± 0.031	Brood care	< 0.001	− 0.103 ± 0.010
Social interaction	< 0.001	0.090 ± 0.013	Walking	< 0.001	0.102 ± 0.013
Food processing	< 0.001	0.085 ± 0.013	Self-maintenance	0.017	− 0.053 ± 0.015
Walking	< 0.001	0.062 ± 0.014	Social interaction	1	–
Self–maintenance	0.006	− 0.048 ± 0.015	Idle	1	–
Brood care	0.053	− 0.021 ± 0.008	Colony maintenance	1	–
Colony maintenance	1	–	Food processing	1	–

*P*-values are Bonferroni-corrected for multiple comparisons. Behaviors are listed in decreasing order of the absolute magnitude of the slope. Shaded boxes highlight behaviors that changed by a magnitude greater than 0.100.

We also measured the distribution of performance across task categories by calculating Shannon’s diversity index (*H*_tasks_) for workers and for queens across pooled queen number treatments and used LMMs to assess the effect of worker number and colony age (fixed effects) on diversity of task performance (*H*_tasks_, response variable), using colony ID as a random effect. Here, *H*_tasks_ is calculated as the total diversity of tasks performed across all four scan samples for all queens or all workers within a single colony on a single sampling day (for details on *H*_tasks_, see^38^). We confirmed normality and homoscedasticity of data with QQ-plots and by plotting fitted values versus residuals, respectively. Models were fitted and evaluated using the “lme4”^49^ and “MuMIn”^53^ packages. Behavioral results are presented as mean estimates ± standard error of GLMM slopes (change in incidence of behavior/worker number).

## Results

### Colony growth and survival

**Figure 1:**
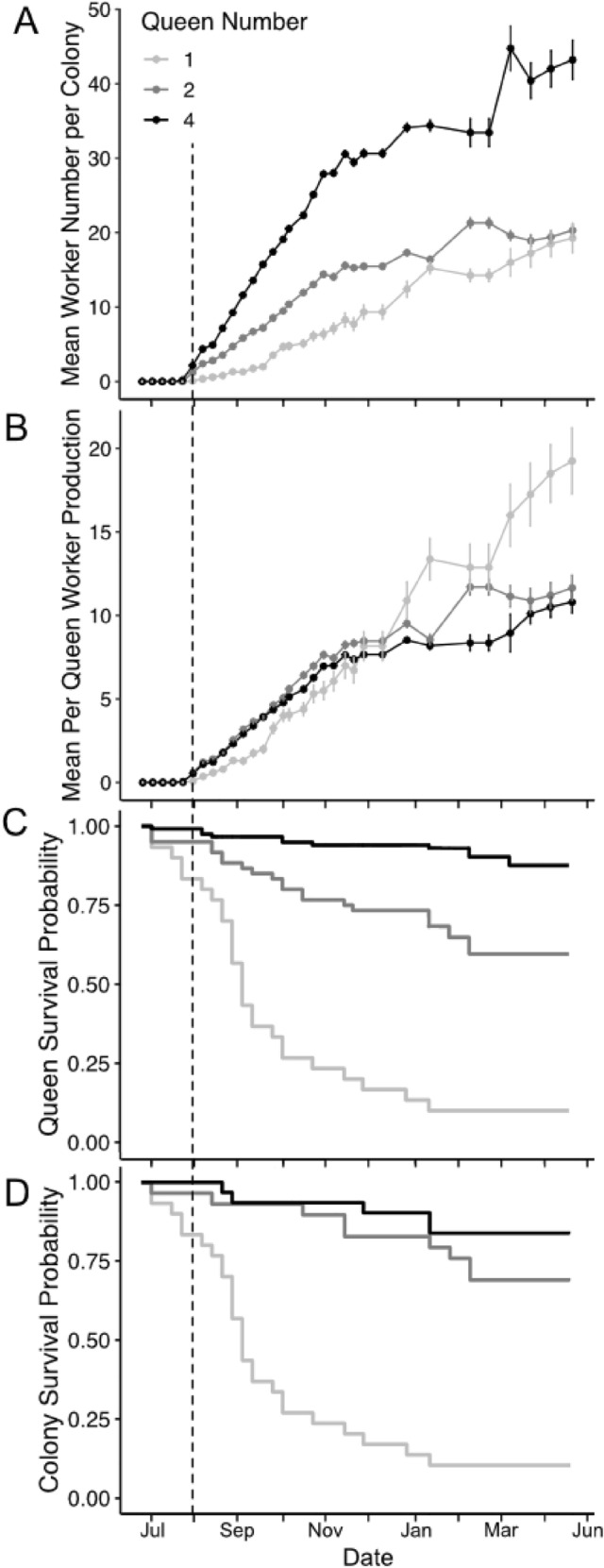
Metrics of colony growth and survival during the first ten months post-founding in colonies initiated by one, two, or four queens, including: (**A**) Colony size (worker number), (**B**) per-queen worker production estimated as the mean worker number divided by the number of queens, (**C**) survival probability of individual queens, and (**D**) survival probability of colonies, where colony survival is defined as the survival of at least one queen. Points and bars represent means and standard errors, and the dashed vertical line marks the week in which the majority of colonies experienced first-worker emergence.

During the first ten months following colony founding on June 26, colonies grew to an average size of 27.58 ± 4.19 workers (Fig. 1A). Over the entire ten months of growth, four-queen colonies grew fastest (GLMM: *P* < 0.001; Post-hoc Tukey tests: 4 vs. 2 queens, *P* = 0.034; 4 vs. 1 queen, *P* < 0.001), reaching an average of 10 workers by day 70 after founding. Two-queen colonies grew faster than single queens (Tukey test: *P* < 0.001), reaching an average of 10 workers by day 102. One-queen colonies grew slowest (Tukey test: *P* < 0.001 for both comparisons), not reaching 10 workers until midway through the winter, on day 184 (Fig. 1A). Although their total worker numbers were higher, multi-queen colonies produced, on average, fewer workers per queen than did single-queen colonies (GLMM: *P* < 0.001; Post-hoc Tukey tests: 4 vs 1 queen, *P* < 0.001; 2 vs. 1 queen, *P* < 0.001). We observed no difference between four- and two-queen colonies in per-queen worker production (Tukey test: 4 vs. 2 queens, *P* = 0.937); (Fig. 1B).

Throughout colony ontogeny, multi-queen colonies had higher survival than single-queen colonies (Log-rank survival analysis: *P* < 0.001; Post-hoc Tukey tests: 1 vs. 2 queens, *P* < 0.001; 1 vs. 4 queens, *P* < 0.001); (Fig. 1D), as measured by the presence of at least one surviving queen. Of the 30 colonies initiated per treatment, by the end of the experiment 26 4-queen colonies, 20 2-queen colonies, and 3 1-queen colonies remained. This is consistent with our expectations, given that multi-queen colonies can lose queens and still remain viable. Survival of colonies founded by two queens versus those founded by four queens did not differ significantly (*P* = 0.560). Furthermore, individual queens were more likely to survive in groups than alone (Mixed-effect cox regression: *P* < 0.001; Post-hoc Tukey tests: 1 vs. 2 queens, *P* < 0.001; 1 vs. 4 queens, *P* < 0.001); (Fig. 1C). Additionally, queens in colonies that were founded by four queens had higher individual survival than those in colonies founded by two queens (*P* < 0.001), indicating a positive effect of cooperation on queen survival.

### Queen behavior during colony ontogeny

**Figure 2:**
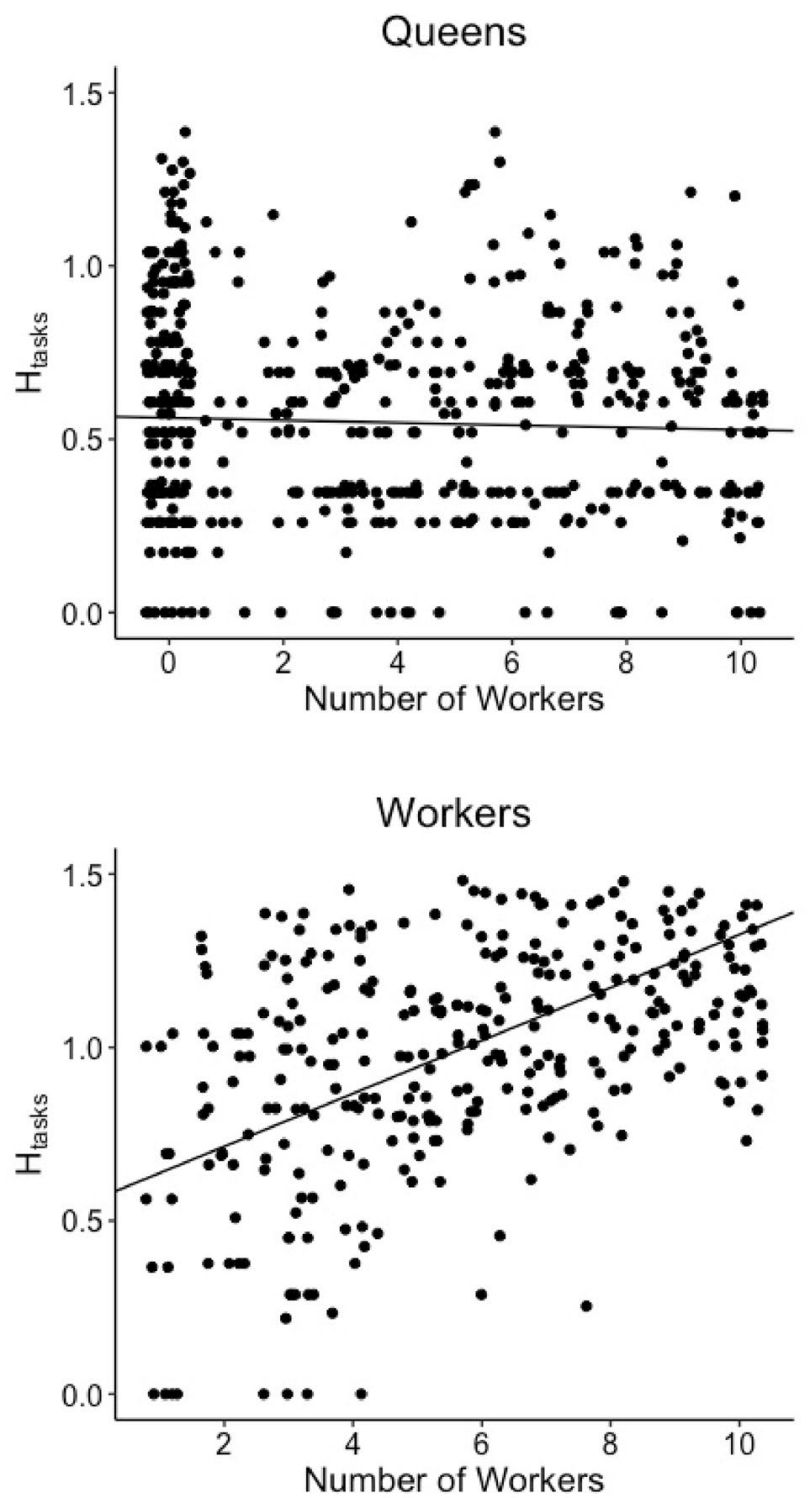
The Shannon Index measuring task performance diversity (H_tasks_) for queens (top) and workers (bottom) for all colonies as a function of number of workers. Queen task performance diversity decreased as worker number increased (LMM, *P* < 0.001, slope = − 0.003, *R*^2^ = 0.116), whereas worker task performance increased with worker number (LMM, *P* < 0.001, slope = 0.076, *R*^2^ = 0.356). Each point represents a single day of scan sampling for a single colony.

**Figure 3:**
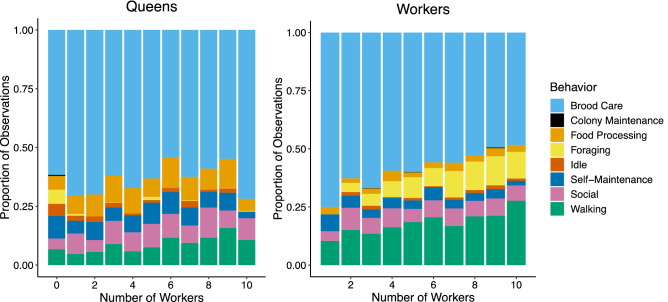
Behaviors of queens (left) and workers (right) represented as the mean proportion of total scan sampling periods in which a given behavior was observed, as a function of colony worker number. As worker number increased, queens spent significantly less time idle and foraging, while workers spent less time on brood care and more time on foraging and walking (GLMM, *P* < 0.001 for all behaviors described here).

The diversity of tasks performed by queens (Shannon Index, *H*_tasks_) slightly decreased as colonies grew larger (LMM, *P* < 0.001, slope = − 0.004, *R*^2^ = 0.116); (Fig. 2) and older (Supplementary Fig. S1). Throughout colony ontogeny, queens spent the majority of time (54–71% of observations) performing brood care tasks. The seven other behaviors occurred infrequently, with each represented in less than 20% of observations (Fig. 3). As the number of workers increased, queens spent significantly less time foraging (GLMM, − 0.544 ± 0.077, *P* < 0.001) and being idle (GLMM, − 0.120 ± 0.031, *P* < 0.001); (Table 1). We also observed significant changes in all other behavioral categories but colony maintenance, but the relative magnitude of these changes was small and may not represent biologically meaningful trends (Table 1).

### Worker behavior during colony ontogeny

Like queens, workers spent the majority of time caring for brood throughout early colony ontogeny. Unlike queens, however, worker task performance diversity increased as colonies grew (LMM, *P* < 0.001; slope = 0.076, *R*^2^ = 0.356), with the magnitude of this increase greater than the corresponding decrease observed in queens (LMM, *P* < 0.001; slope = − 0.003); (Fig. 2). As worker number increased, workers spent less time on brood care (GLMM,—0.103 ± 0.010, *P* < 0.001), and more time on foraging (GLMM, 0.134 ± 0.018, *P* < 0.001) and walking (GLMM, 0.102 ± 0.013, *P* < 0.001). We also observed a significant but minor decrease in time spent on self-maintenance (GLMM,—0.053 ± 0.015, P = 0.017); (Fig. 3); (Table 1). We detected no effect of queen number on the incidence of any of these worker behaviors (Supplementary Table S1).

## Discussion

Intrinsic costs of cooperating with non-relatives suggest that non-kin sociality may arise as an adaptation to harsh or challenging conditions^7,54,55^. For eusocial groups, early colony ontogeny marks a particularly challenging life history stage accompanied by major changes in queen and worker labor roles^8–10^. Our results demonstrate persistent survival and growth advantages of queen cooperation during the phase of transition from pleometrosis during colony founding to primary polygyny during early colony growth by the harvester ant *P. californicus*. These important benefits can favor long-term cooperation even at the expense of reduced reproductive output at maturity^7^, suggesting a role for ontogenetic challenges in facilitating the evolution of non-kin cooperation.

### Survival and growth advantages of cooperative founding during early colony ontogeny

Across animal taxa, cooperation during breeding is an important adaptation to harsh and risky environments^56–62^. Likewise, for eusocial insects, cooperation among foundresses (pleometrosis) is a major strategy for mitigating the risks and costs of colony founding^14,22,23,28^. In most pleometrotically-founding ants, these cooperative associations become antagonistic shortly after first worker emergence, as within-group fighting reduces the queen number to one^24,27,63^. Rarely, however, multiple queens can persist throughout the lifetime of the colony (primary polygyny)^27,44^, suggesting prolonged benefits of cooperation past founding and through colony ontogeny, particularly in relation to ecological context and competition. In the case of *P. californicus*, resource limitation and intraspecific competition may drive queen cooperation in polygynous populations, even though individual reproductive output is generally lower for polygynous queens^7^. Though queens can benefit throughout the colony lifespan from the production of a genetically diverse workforce^64–68^, high levels of polyandry in *P. californicus* suggest that additional queens may not consequentially increase already-high worker genetic diversity^44^. Instead, our results demonstrate that cooperation in this context may be driven by persistent survival and growth advantages during the period of transition from pleometrosis to primary polygyny.

Multi-queen colonies in our study had higher survival rates than did single-queen colonies. Importantly, increased colony survival was not merely a product of the availability of replacement queens in the event of queen death; queens also had higher individual survival when founding nests cooperatively. Survival advantages have been demonstrated for several species forming pleometrotic associations^14,21–23,28^, but in this study, we show that this advantage extends months past the first emergence of workers, supporting the long-term retention of queens seen in primary polygyny. Furthermore, the sharpest decline in single-queen survival occurs several weeks after first worker emergence, suggesting that the greatest advantages of queen cooperation in these populations may occur post-founding.

It is not yet clear what causes this differential mortality between the cooperative and solitary-founding conditions, but it is likely that queens benefit from shared labor that reduces the average personal investment in risky and/or costly tasks. Foraging, in particular, is both risky and energetically costly, but these risks and costs are minimized for lab-reared colonies that face no predators and travel a distance less than ten centimeters to gather food. Excavation likewise poses physiological costs, especially through cuticular abrasion caused by contact with dirt, which increases rates of water loss^69^. However, our experimental set-up did not provide dirt for excavation, suggesting that this task does not wholly account for the increased mortality of single queens. Likewise, although food limitation may favor polygyny in field colonies^7^, we found that multi-queen colonies experienced survival advantages over single-queen colonies even under ad libitum feeding conditions. One interesting possibility is that social behaviors such as allogrooming reduce mortality by improving pathogen removal efforts^70–72^. Future work should disentangle the suite of potential proximate benefits that contribute to this social survival advantage.

Queens of young colonies likewise may benefit from reduced individual brood production costs in pleometrotic associations^21^. Our results demonstrate that cooperatively founding queens experienced faster colony growth and larger colony sizes within the first ten months of ontogeny. Rapid growth speeds colonies past the riskiest stages of ontogeny by assembling a robust workforce at the time when young colonies are most vulnerable to mortality and predation^9^. Additionally, we found that throughout colony ontogeny queen groups produce fewer workers per queen on average than do single queens. By sharing the task of sterile worker production, cooperative queens may reap important energy savings associated with egg production. The increased worker production efficiency of groups may have important implications for longevity and/or fitness at colony maturity.

### Shifts in organization of labor during early ontogeny

Growth by several orders of magnitude presents a labor scaling problem: how does a small group cope with rapid growth to states where existing work organization programs are no longer applicable, productive, or efficient? The problem of labor scaling is common in human social issues, particularly in the context of urban growth and economic productivity^73,74^. Likewise, a major problem in computer systems is the scaling of servers or processes to optimize performance across computing loads^75,76^. In biological systems, organisms (and groups of organisms) similarly face the challenge of scaling work following growth by several orders of magnitude. Our results indicate that the scaling of work organization during colony ontogeny in *P. californicus* occurs not through an abrupt reprogramming of labor roles, but instead through a gradual reallocation of tasks.

We found that growing colonies of *P. californicus* redistribute labor from queens to workers throughout early ontogeny. As colonies grew to a size of ten workers, the diversity of tasks performed by queens decreased slightly, perhaps reflecting a shrinking task repertoire at the transition from foundress to queen. Simultaneously, workers increased their task performance diversity with worker number. These parallel, simultaneous shifts suggest that the increasing task repertoire of workers enables queens to abandon high-cost tasks associated with founding. Indeed, the largest magnitude change in queen behavior was observed in foraging, which decreased in frequency as workers emerged. *P. californicus* queens are semi-claustral, meaning that they lack sufficient physiological reserves to remain in the nest during colony founding, and obligately forage to support the development of their first brood^77^. Simultaneously, workers increased their frequency of foraging as colonies grew, suggesting a transfer of risky and/or expensive labor from valuable queens to relatively expendable workers. The prolonged investment by queens in non-reproductive tasks during early ontogeny enables the gradual upward scaling of work by young offspring^27,77–79^.

As workers increased their time spent foraging, they similarly increased their time spent walking in the nest. Although this behavior is not often assigned to a functional task category^38^, it is a useful indicator of worker transit between discrete task-associated areas of the nest, such as the brood pile or seed cache. Walking may also serve to stimulate encounters between workers, ensuring information flow within the nest, and to promote contact with task-associated stimuli. These functions all support a role for walking in the diversity of task performance, which increased as colonies grew. Correspondingly, we observed a substantial decrease in time spent idle (inactive away from brood) by queens, combined with a minor but significant increase in walking.

Given the pressing need for growth at this stage of colony ontogeny, brood care made up the majority of tasks performed by both queens and workers. Workers, however, substantially decreased their performance of brood care as colonies grew. This decrease suggests an increasing need to perform a broader diversity of growth and maintenance-related tasks when colony size increases. This pattern is also consistent with changes in behavior expected under age polyethism, where young workers specialize on within-nest activities such as brood care, and older workers specialize on beyond-nest activities such as foraging^27,80^. The newly-emerged first worker cohort is likely to perform the brood care tasks associated with their age before venturing outside the nest, accounting for the high frequency of brood care in small, young colonies. However, this trend may shift later in ontogeny: Holbrook et al*.*^38^ found that young colonies of 10–30 workers performed significantly more brood care than they did when they reached several hundred workers. In the context of previous findings, these dynamic changes in task allocation may reflect a general progression from investment in survival during founding, to growth after first worker emergence, to maintenance after colony establishment.

## Conclusions

Cooperation among non-kin presents an evolutionary puzzle, in which indirect fitness benefits are insufficient to explain cooperative behavior. Primary polygyny provides a useful model for understanding the conditions that favor non-kin cooperation despite substantial direct fitness costs of shared reproduction^7^. We observed significant survival and growth advantages of primary polygyny in *P. californicus* during early colony ontogeny, a transitional stage marked by gradual shifts in the allocation of labor by queens and workers. Polygynous colonies accelerated quickly through this transition with faster colony growth, despite lower per-queen worker production rates. Further, polygynous queens experienced improved survival even in the absence of important founding stressors such as food limitation, physiological costs of excavation, and risks of predation. The advantages of cooperation under these conditions suggest the importance of alternative factors, especially brood production costs, for explaining the adaptive value of polygyny during colony ontogeny. Importantly, these survival and growth benefits outlast the founding period, providing critical advantages during a challenging life history stage and favoring selection for cooperative behavior that is stable rather than ephemeral.

## Supplementary Information

Supplementary Information.
