# Supplementary material for: Cooperation among unrelated ant queens provides persistent growth and survival benefits during colony ontogeny
Source: Sci Rep. 2021 Apr 15;11:8332. doi: 10.1038/s41598-021-87797-5 (PMC8050306; doi:10.1038/s41598-021-87797-5)
Supplement: Supplementary file 1 — Supplementary Information. [file 41598_2021_87797_MOESM1_ESM.docx]

**Supplementary Materials**

**Cooperation among unrelated ant queens provides persistent growth and survival benefits during colony ontogeny**

Madeleine M. Ostwald*, Xiaohui Guo, Tyler Wong, Armon Malaekeh, Jon F. Harrison, and Jennifer H. Fewell

School of Life Sciences, Arizona State University, Tempe, AZ, USA

*corresponding author

Corresponding author email: mostwald@asu.edu

Supplementary Table 1: Summarized results of GLMM predicting worker behavior as a function of worker number, queen number, and the interaction between queen and worker number (fixed effects), with colony ID as a random effect. *P*-values are Bonferroni-corrected for multiple comparisons. No significant effects of queen number or the interaction effect prompted us to remove them from the model reported in Table 1.

|  | ***P*-value** | |
| --- | --- | --- |
| **Behavior** | **Effect of Queen Number** | **Effect of Interaction Between Queen Number and Worker Number** |
| **Brood Care** | 0.164 | 0.617 |
| **Social** | 1 | 0.646 |
| **Idle** | 1 | 1 |
| **Colony Maintenance** | 1 | 1 |
| **Walking** | 0.600 | 1 |
| **Food Processing** | 0.918 | 1 |
| **Foraging** | 1 | 0.815 |


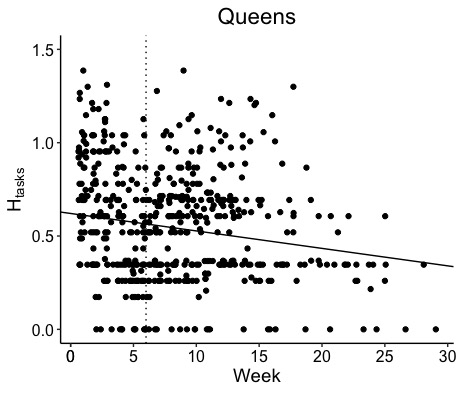


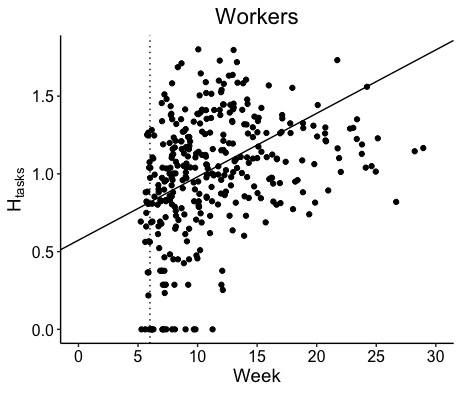


Supplementary Figure 1. The Shannon Index measuring task performance diversity (H_tasks_) for queens (top) and workers (bottom) for all colonies measured until the tenth worker emerged. Queen task performance diversity decreased over time (LMM, *P* < 0.001, slope = -0.009, *R*^2^ = 0.157), whereas worker task performance increased over time (LMM, *P*<0.001; slope = 0.041, *R*^2^ = 0.358). Dashed vertical lines mark the week in which the majority of colonies experienced first-worker emergence.
